# Bioinformatic discovery of a toxin family in *Chryseobacterium piperi* with sequence similarity to botulinum neurotoxins

**DOI:** 10.1038/s41598-018-37647-8

**Published:** 2019-02-07

**Authors:** Michael James Mansfield, Travis Gwynn Wentz, Sicai Zhang, Elliot Jeon Lee, Min Dong, Shashi Kant Sharma, Andrew Charles Doxey

**Affiliations:** 1 Department of Biology, University of Waterloo, 200 University Ave. West, Waterloo, Ontario N2L 3G1 Canada; 2 Division of Microbiology, Center for Food Safety and Applied Nutrition, United States Food and Drug Administration, College Park, MD 20740 USA; 3 Department of Urology, Boston Children’s Hospital, Department of Microbiology and Immunobiology and Department of Surgery, Harvard Medical School, Boston, MA 02115 USA

## Abstract

Clostridial neurotoxins (CNTs), which include botulinum neurotoxins (BoNTs) and tetanus neurotoxin (TeNT), are the most potent toxins known to science and are the causative agents of botulism and tetanus, respectively. The evolutionary origins of CNTs and their relationships to other proteins remains an intriguing question. Here we present a large-scale bioinformatic screen for putative toxin genes in all currently available genomes. We detect a total of 311 protein sequences displaying at least partial homology to BoNTs, including 161 predicted toxin sequences that have never been characterized. We focus on a novel toxin family from *Chryseobacterium piperi* with homology to BoNTs. We resequenced the genome of *C*. *piperi* to confirm and further analyze the genomic context of these toxins, and also examined their potential toxicity by expression of the protease domain of one *C*. *piperi* toxin in human cells. Our analysis suggests that these *C*. *piperi* sequences encode a novel family of metalloprotease toxins that are distantly related to BoNTs with similar domain architecture. These toxins target a yet unknown class of substrates, potentially reflecting divergence in substrate specificity between the metalloprotease domains of these toxins and the related metalloprotease domain of clostridial neurotoxins.

## Introduction

Clostridial neurotoxins (CNTs), including botulinum neurotoxins (BoNTs) and tetanus neurotoxin (TeNT), respectively, are the causative agents of botulism and tetanus and are the deadliest known biological toxin family with LD_50_ values ranging from 0.1–1.0 ng per kg^1^. Owing to their extreme toxicity, BoNTs are potential bioterrorism agents, and yet also have enormous utility as protein therapeutics^2,3^. BoNTs are produced by *Clostridium botulinum*, a polyphyletic taxon classified solely by the presence of the neurotoxin, and several other species of *Clostridium*. Neurotoxin genes reside in distinct gene clusters encoded on the chromosome, plasmids or phages. All BoNTs are neighbored by the NTNH (non-toxic non-hemagglutinin) gene, which encodes a homolog of BoNT that lacks the HExxH motif and forms part of the progenitor toxin complex. There are currently seven universally accepted, antigenically distinct BoNT serotypes, designated BoNT/A-G, as well as several recombinant mosaics (C/D, D/C, and F5A). A new BoNT serotype (BoNT/X) has been recently identified in the genome sequence of *C*. *botulinum* strain 111^4^. A subtype numeral (e.g. BoNT/A1) is also designated to label a growing number of divergent sequences within serotypes^5^.

The extreme toxicity of BoNT is a consequence of its unique structure and function (Supplementary Fig. S1). BoNTs are initially produced as a single polypeptide chain, which is then cleaved by bacterial or host proteases to result in a light-chain (LC) and heavy-chain (HC) which remain linked by a disulfide bond. The HC contains two functional domains: the N-terminal translocation domain (H_N_) and the C-terminal receptor-binding domain (H_C_). The receptor-binding domain can be further divided into two subdomains, consisting of an N-terminal laminin-like jelly roll fold (H_CN_) and a C-terminal ricin-type beta-trefoil fold (H_CC_). BoNTs recognize motor nerve terminals by targeting neuronal receptors, including SV2 for BoNT serotypes A/D/E/F, and synaptotagmin I/II for BoNT serotypes B/G/DC, with polysialogangliosides as co-receptors^6–17^. After neuronal binding, BoNTs are internalized within endocytic vesicles. At low pH, the H_N_, which forms an all alpha-helical bundle structure, transports the partially unfolded LC into the cytosol. The LC, composed of a ~400 residue N-terminal zinc metalloprotease domain, then cleaves intracellular SNARE proteins including VAMPs, SNAP25, and syntaxin 1^18–21^ to prevent exocytosis of synaptic vesicles, resulting in flaccid paralysis^22^.

Recent work by Mansfield *et al*. (2015) reported a divergent BoNT homolog in the genome of *Weissella oryzae*, which suggested that BoNT-related proteins are not limited to the genus *Clostridium*^23,24^. This hypothesis has been further supported by the recent discovery of BoNT/En, a novel BoNT in *Enterococcus faecium* strain IDI0629^25,26^, which was demonstrated to cleave both SNAP25 and VAMP2^25^. The presence of BoNT homologs in *Weissella* and *Enterococcus* raises the intriguing possibility that a larger family of BoNT-related toxins exists in a broader range of bacterial taxa^27^. These homologs may include not only toxins with globally conserved domain architectures, but potentially distant homologs of BoNTs with more divergent domain architectures, sequences and functions^27–29^.

Here we present a large-scale bioinformatic screen for putative toxin genes in all currently available genomes. Unlike previous studies, we did not limit our searches to the detection of complete homologs, but also considered detectable similarities involving individual BoNT domains to increase the chance of detecting distant homologs. Our analysis identified hundreds of putative toxins, and revealed a novel toxin family from *Chryseobacterium piperi*^30^ that exhibits distant homology to BoNTs and has a similar domain architecture. We resequenced the genome of *C*. *piperi* to confirm and further analyze the genomic context of these toxins, and also examined their potential toxicity by transfection assays into human cells. These toxins target a yet unknown class of substrates, potentially reflecting divergence in substrate specificity between the metalloprotease domains of these toxins and the related metalloprotease domain of clostridial neurotoxins.

## Results

### Genomic data mining uncovers proteins with BoNT-like domains

We screened the NCBI GenBank database (March 26, 2017) comprised of 94,396 prokaryotic, 4,123 eukaryotic, and 7,178 viral genomes, for potential homologs of BoNTs over one or more domains. Using PSI-BLAST with selected BoNT sequence queries (see Methods), we detected a total of 311 protein sequences displaying at least partial homology to BoNTs with an *E*-value below 0.001 (Fig. S1a, Table S1). The dataset includes all known BoNT serotypes, and an additional 161 predicted toxin sequences, all of which are experimentally uncharacterized to date.

**Figure 1:**
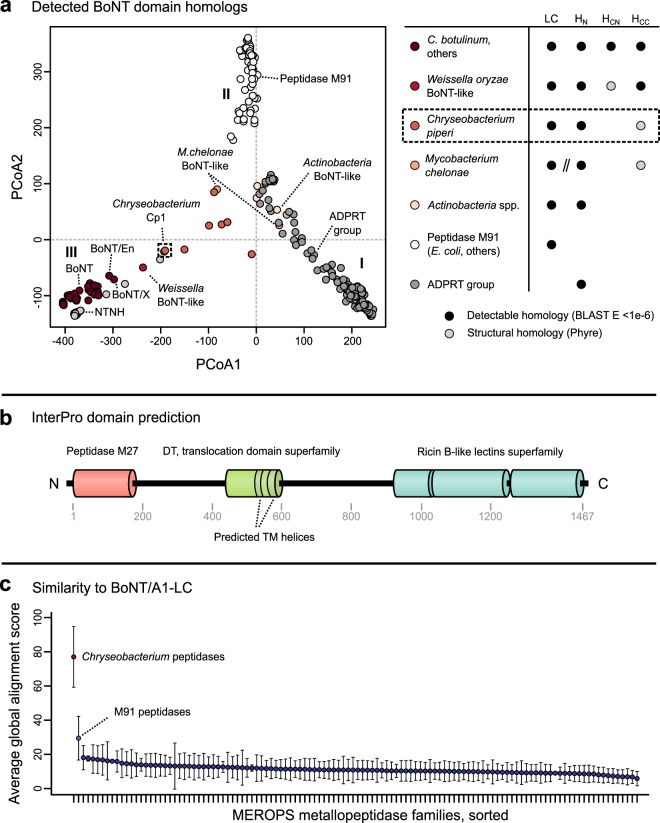
Bioinformatic detection of BoNT-related genes in microbial genomes. (**a**) PCoA ordination of pairwise percent similarities reveals relationships between the sequence families surveyed in this study. A large family of ADP-ribosyltransferase toxins, including diphtheria toxin-like proteins and a large family of predicted toxins from entomopathogenic fungi possess similarity only over the translocase domain (cluster I). M91 peptidases group separately (cluster II). BoNT and NTNH form distinct groups, with more divergent relatives such as the *Weissella* BoNT-like protein clustering outside them. The next closest relatives are the BoNT-like toxins found in *Chryseobacterium*, followed by putative toxins from other *Actinobacteria* (cluster III), which span the distance between groups, having similarities to each. Notably, genes encoding *M*. *chelonae* BoNT-like proteins appear to be split into two components (one encoding the “light chain” M27-like peptidase, and the second encoding the “heavy chain” translocation domain and receptor-binding domain). (**b**) InterPro domain predictions for *Chryseobacterium piperi* BoNT-like protein Cp1 (WP_034687872.1), revealing a similar architecture to BoNT. The translocase domain is annotated as diphtheria-like, and contains two predicted transmembrane helices. (**c**) Comparison of BoNT/A1 peptidase domain to *C*. *piperi* putative peptidases and peptidases in the MEROPS database. Except for peptidase M27 (not pictured), the peptidases from *Chryseobacterium* produce the highest-scoring global alignments, followed by peptidase M91. Both *C*. *piperi* peptidases and M91 peptidases score higher than all other known peptidase families.

We performed all-by-all pairwise alignments and clustered the toxins using principal coordinates analysis (PCoA). The toxins clustered largely into three main groups, which differ in domain composition and detectable similarities to BoNTs (Fig. 1a). Group I includes a large family of ADP-ribosyltransferase toxins, including diphtheria toxin-like sequences^31^ and putative ADP-ribosyltransferase toxins (ADPRTs) from entomopathogenic fungi (Table S1). These sequences possess partial similarity only to the BoNT translocation domain (17.3% maximum sequence identity, PSI-BLAST *E*-value = 7 × 10^−40^). Group II is formed by M91 family peptidases such as the *Escherichia coli* type III effector toxin NleD, which cleaves host JNK and p38^32^. These sequences possess remote detectable homology to the BoNT-LC with 14.9% maximum sequence identity and a PSI-BLAST *E*-value of 4 × 10^−5^ (see Methods).

Group III contains BoNTs, NTNHs, the *Weissella* toxin and several uncharacterized proteins (Fig. 1a) that share multiple domains in common with BoNTs (Fig. S2) and are therefore of considerable interest^23,33^. Among the uncharacterized proteins are nine partial and full-length homologs from the genome of *Chryseobacterium piperi*, two from *Mycobacterium chelonae*, and five from other *Actinobacteria*. Several of these organisms are associated with disease; some *Chryseobacterium* species are known opportunistic pathogens^34,35^, *Acaricomes phytoseiuli* is a pathogen of mites^36^, and *Mycobacterium chelonae* is a human pathogen associated with skin, soft tissue, and bone infections^37^. We termed these proteins “divergent BoNT homologs” given their distant but significant detectable evolutionary relationship to BoNTs (Fig. 1a) and similarity of domain architecture (Fig. 1b). As shown for a representative protein from this group (putative *Chryseobacterium* toxin, “Cp1”, NCBI accession number WP_034687872.1), these proteins are predicted by InterProScan^38^ to contain a BoNT-like three domain architecture composed of a metalloprotease domain, central translocation domain and a C-terminal ricin-type beta-trefoil domain (Fig. 1b), each of which are analyzed in greater detail below. Cp1 for example possesses detectable homology to BoNTs spanning multiple domains (Fig. S2), but has low sequence identity (17% identity between Cp1 and BoNT/A compared to >=28% identity between BoNT family members) indicative of a distant evolutionary relationship.

To further confirm this detected relationship, we compared BoNT/A1-LC to the *C*. *piperi* toxin peptidase domains, as well as all known metallopeptidase families from the MEROPS database^39^, consisting of 220,362 sequences from 102 families (Fig. 1c). Based on alignment scores, the *C*. *piperi* toxins displayed stronger similarities to BoNT-LC than to all other known protease families, and the M91 protease family ranked second.

### *Chryseobacterium* toxins are a novel toxin family distinct from but related to BoNTs

**Figure 2:**
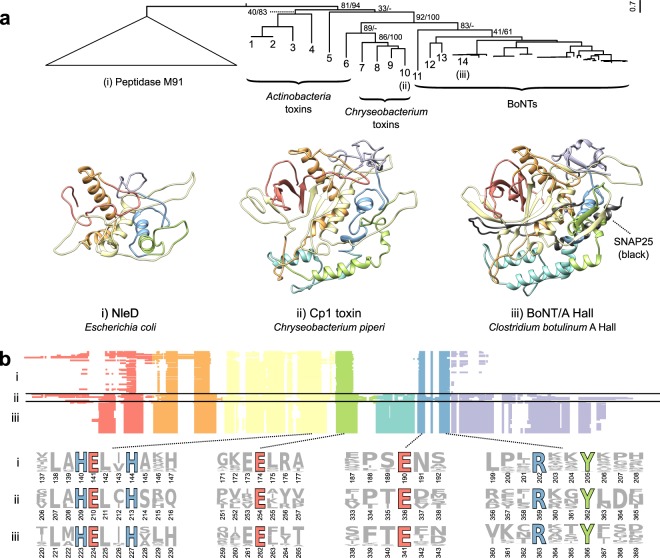
Comparison of the BoNT-LC with homologous domains from BoNT-like toxins and M91 family proteases. (**a**) Phylogenetically, BoNTs and divergent BoNT homologs group distinct from distantly related peptidase M91 sequences. Statistical support for the tree is indicated as maximum likelihood bootstrap value and Bayesian posterior probability (percentage), respectively. Structural comparison of BoNT/A (PDB 1XTG) (iii) with structural models of *Chryseobacterium* Cp1 (ii) and *E*. *coli* NleD (i) reveals two regions that are unique to BoNTs and BoNT-like proteins: the lower alpha-helical region, which interacts directly with SNARE substrates, and the C-terminal region that plays a role in catalytic product removal. (**b**) M91 family peptidase domains (i), divergent BoNT homologs (ii), and BoNT-LCs (iii) have key conserved sequence features. These include the HExxH zinc-coordinating and catalytic residues, the third zinc ligand E261, and the active site-refining E350 and RxxY motif. As depicted in the multiple alignment, two insertion regions appear unique to BoNTs and divergent BoNT homologs, shown in teal and purple respectively. The identities of proteins labeled 1-14 are: 1 – WP_037712107.1, *Streptomyces* sp. AA4; 2 – EFL04418.1, *Streptomyces* sp. AA4; 3 – WP_083906476.1, *Acaricomes phytoseiuli*; 4 – GAO13068.1, *Streptomyces* sp. NBRC_110027, 5 – WP_055473237.1, *Streptomyces pathocidini*; 6 – WP_070931163.1, *Mycobacterium chelonae*; 7 – WP_034681281.1, *Chryseobacterium piperi*; 8 – WP_034687877.1, *Chyrseobacterium piperi*; 9 *-* WP_034687193.1, *Chryseobacterium piperi*; 10 – WP_034687872.1, *Chryseobacterium piperi*; 11 – WP_027699549.1, *Weissella oryzae*; 12 – WP_086311652.1, *Enterococcus* BoNT/En; 13 – BAQ12790.1, BoNT/X; 14 – ABS38337.1, BoNT/A1.

Next, the alignment of protease domains from BoNTs and the divergent BoNT homologs was analyzed further to perform sequence, structural, and phylogenetic analysis (Fig. 2). Phylogenetically, BoNT-LCs grouped into a distinct clade, with BoNT/X and BoNT/En forming divergent early branching lineages (Figs 2a and S3). The BoNT clade is outgrouped by lineages consisting of the predicted toxins from *Weissella*, *Chryseobacterium*, and *Mycobacterium*, although the precise branch order is difficult to resolve with the available data. Nonetheless, the BoNTs together with the *Weissella*, *Mycobacterium*, and *Chryseobacterium* toxins form a distinct clade from the peptidase M91 group with high statistical confidence (83% maximum likelihood bootstrap support and 100% Bayesian posterior probability) (Figs 2a and S3). Protease domains from the actinobacterial toxins group more distantly, and the clade of distantly related M91 family proteases forms a lineage distinct from BoNTs and the divergent BoNT homologs (Fig. 2a). Despite some variable segments and low sequence identity (BoNTA1/Cp1: 17.6%), the protease domains from *C*. *piperi* and other divergent BoNT homologs possess detectable homology to the BoNT-LC (bl2seq *E*-value = 2 × 10^−6^ between Cp1 and BoNT/A1) and conserve key functional residues found in BoNTs (Fig. 2b). These residues include: the critical HExxH zinc-coordinating active site motif; the third zinc ligand Glu-261; the functionally important Glu-350 which shapes active site fine structure, the active site stabilizing motif R363-x-x-Tyr366^40^, and the cysteine residues that form the disulfide bridge between BoNT LC and HC^41^ (Fig. 2b).

Consistent with phylogenetic analysis, the predicted structure of the protease domain of *C*. *piperi* toxin is most similar to BoNT-LC (7.0 Å RMSD versus 12.0 Å for *E*. *coli* NleD; both models based on BoNT template structures) (Fig. 2a). Although experimental structure determination is required to confirm these models, several obvious structural differences can be inferred based on the models and sequence alignments. One insertion is common to BoNT-LCs and the divergent BoNT homologs, and absent in NleD and other M91 peptidases (Fig. 2a,b), which makes extensive contacts with SNAP25 (51 inter-residue contacts <2 Å) and VAMP2 (91 inter-residue contacts <2 Å) in BoNT co-crystal complexes (Fig. S4). This insertion may therefore have contributed to an ancestral shift in substrate specificity between M27 and M91 peptidase families. A second C-terminal insertion common to BoNT-LC and the divergent BoNT homologs (Fig. 2a,b) forms part of the hydrophobic SNAP25 binding pocket^42^, and has been shown to mediate catalytic activity and product removal^43^. Lastly, a region corresponding to the “belt” region present in BoNTs was identified in *C*. *piperi* toxins based on multiple sequence alignment, but this region did not display significant sequence similarity to BoNT, suggesting that it is highly divergent or unrelated.

**Figure 3:**
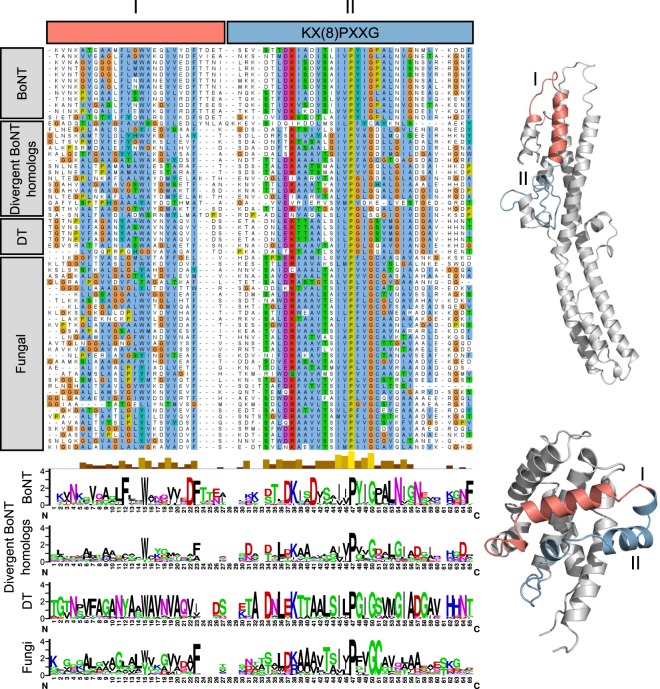
Translocase similarity between BoNTs, BoNT-like toxins and diphtheria toxins. A subdomain of BoNT translocases contains significant similarity to diphtheria T domains (DT in the above) as well as the translocase region of BoNT-like toxins. One segment of similarity (I) corresponds to the transmembrane TH5 helix in diphtheria toxins and a loop in BoNTs. A second region (II), including the key K(x)8PxxG motif, is also strongly conserved. For ease of visualization, only a portion of the translocase alignment has been pictured here.

In addition to LC conservation, *Chryseobacterium* and other divergent BoNT homologs possess significant similarity to the BoNT translocation domain (22% identity, bl2seq *E*-value < 1 × 10^−5^), particularly across the region 593–686 in BoNT/A1 (Fig. 3), which has been suggested to form a channel-forming amphipathic alpha helical motif^44–46^. Unexpectedly, BLAST searches of this region also detected a segment of the diphtheria toxin (DT) translocation domain (residues 286–325, helices TH5-TH6/TH7, PDB ID 4AE0)^47^, which was consistent with structural predictions for this region made by PHYRE (Fig. S2). Sequence alignment revealed a common region of sequence similarity flanking a motif ([K/R]x(8)PxxG) within the translocation domains of the divergent BoNT homologs, BoNT, and DT (Figs 1b and 3). Although the functional significance of this shared motif is unclear, the detectable similarity to both BoNT and DT translocation domains strongly suggests a translocation-related function for this region in *C*. *piperi* toxin.

Lastly, following the translocation domain, the divergent BoNT homologs possess a receptor-binding domain that is predicted to adopt the same fold as the BoNT H_CC_ domain (Figs 1b and S2). A ricin-type beta-trefoil fold was predicted for the C-terminal region of the putative *C*. *piperi* toxins by three separate methods (HHpred, Pfam, and Phyre with *E*-values < 0.001). The beta-trefoil domains from *C*. *piperi* toxins exhibited at most 17% sequence similarity to the H_CC_ in BoNT; therefore, the extent to which they are homologous is unclear at this point. Interestingly, a ricin-type beta-trefoil domain from the *C*. *botulinum* hemagglutinin, HA33, was identified as the top template by PHYRE (Fig. S2), indicating that if this domain is not related to the H_CC_ in BoNT, it may be homologous to other ricin-type beta-trefoils that are encoded within BoNT gene clusters.

### Genome resequencing of *C*. *piperi* confirms presence of toxin gene clusters

**Figure 4:**
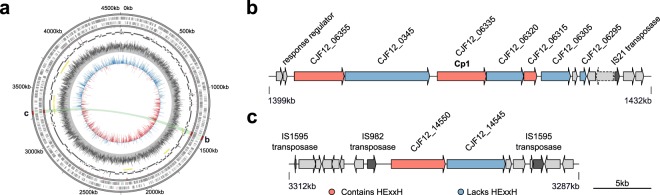
BoNT-like toxins reside within two toxin gene clusters in the genome of *Chryseobacterium piperi*. (**a**) Re-sequencing with a combination of Illumina and PacBio resulted in a closed genome with a single circular chromosome. Yellow bands reflect the local alignment of the toxin containing contigs (shown in **b** and **c**) from the initial JPRJ01 assembly against an intermediate assembly (black), and the closed genome (CP023049). Annotated insertion sequences (red ticks), SRX3229522 read mapping to CP023049 (gray histogram), and GC skew (blue/red histogram) are also indicated. The closed genome contains two separate toxin gene clusters (shown in **b** and **c**). The chromosomal loci that contain the toxin gene clusters have several notable features. First, there is evidence of gene duplication and pseudogenization among toxin genes, including an apparent split of a BoNT-like gene homolog into two genes, consisting of an HExxH-positive peptidase domain (CJF12_06315) and a ‘heavy chain’ coding sequence containing a diphtheria-like translocase and a C-terminal ricin-type beta trefoil domains (CJF12_06305). Genes neighbouring the toxin gene cluster include putative response regulator genes (e.g., CJF12_06360, CJF12_06365, CJF12_06370, CJF12_06375), and several different types of transposases (e.g., CJF12_06285, CJF12_06290, CJF12_14525, CJF12_14555, CJF12_14620). These genes may potentially have a role in the expression or lateral transfer of these toxin gene clusters. Full gene annotations for the clusters are available in the Supplementary Information.

The putative *C*. *piperi* toxins are located on three separate contigs (2, 44, and 59) in the original draft *C*. *piperi* genome (NCBI accession JPRJ01, 89 total contigs). To verify a *C*. *piperi* origin for these contigs, determine extrachromosomal content, and enable further genome-wide analysis, *C*. *piperi* was acquired from ATCC and sequenced on Illumina MiSeq and Pacific Biosciences RS-II sequencing platforms. A closed genome 4.5Mbp in length, 35.3% GC content, and 250X coverage was produced and analysed (Fig. 4). No plasmids were observed. The assembly revealed a toxin gene cluster (GC1) located at 1399–1432 kbp which contains the Cp1 gene as well as 6 other genes with detectable similarity to BoNTs and an alternating pattern of presence/absence of the HExxH motif (Fig. 4, Table S2). A second toxin gene cluster (GC2) is located at 3287–3312 kbp containing two additional genes with detectable similarity to BoNTs (Fig. 4). The genes located in this region have similarity to BoNT over different regions, and may contain or lack the catalytic HExxH motif. Similar to *bont* gene clusters, HExxH-positive homologs in *C*. *piperi* are flanked by genes that also possess detectable partial homology to BoNTs, but lack the active site motif. The paralogous nature and genomic arrangement of these gene pairs resemble that of *ntnh* and *bont*, which raises the possibility that the HExxH-negative genes may play a similar role to *ntnh* in *bont* gene clusters. Further, these HExxH-negative proteins uniquely contain an IBC1 (“Isoprenoid_Biosyn_C1 super family”) domain at the N-terminus similar to class I terpene-synthases, whose role is unclear. No additional neurotoxin-associated genes from the *ha/p47/orfx* families are present in these clusters.

Several genomic features surrounding the *C*. *piperi* toxin gene clusters indicate an origin via mobile element insertion. First, homologous regions to GC1 and GC2 were not detected in any other available *Chryseobacterium* genomes, suggesting non-*Chryseobacterium* origins. Second, numerous transposases are present including two IS110 family transposases, a IS200/IS605 transposase 18–40 kbp upstream (CJF12_06430, CJF12_06460, CJF12_06500), and an IS1182 family transposase (CJF12_05985) 30 kbp downstream of GC1. IS110 transposases have been previously shown to flank other *bont* gene clusters^48^. A disrupted IS982 family transposase pseudogene (CJF12_14555) is located immediately upstream of CJF12_14550 and flanking GC2 are complete and partial IS1595 family, ISChpi, insertion sequences (CJF12_14525 and CJF12_14620). Third, genes neighboring *Chryseobacterium* toxin gene clusters were found to possess homology to genes in *M*. *chelonae* (e.g., closest homolog of CJF12_14560 was *M*. *chelonae* WP_064393402.1, 71% amino acid identity), consistent with the detected similarity between the *C*. *piperi* toxins and *M*. *chelonae* genes (Fig. 2a).

### *C*. *piperi* toxin is a novel metalloproteolytic toxin that induces necrotic cell death

Given the substantial sequence variation between predicted *C*. *piperi* toxins, we selected WP_034687872 (Cp1) for experimental characterization based on it having the greatest sequence similarity to BoNTs among the *C*. *piperi* toxins over catalytic and functional sites (around 35% amino acid similarity). Initial protease assays of recombinant Cp1-LC against known BoNT substrates, including syntaxin 1, VAMP2, and SNAP25, yielded negative results (Fig. S5). Although Cp1-LC did not display activity against canonical BoNT targets, the conservation of the active site residues and similarity to M27 and M91 metallopeptidases suggested the possibility of other targets. We elected to test for broad, metallopeptidase-induced toxicity via transfection and subsequent expression of the Cp1 LC cDNA in human embryonic kidney HEK293T cell line. Two Cp1-LC mutants containing point mutations at the HExxH motif (H209A and E210Q), were utilized as negative controls.

**Figure 5:**
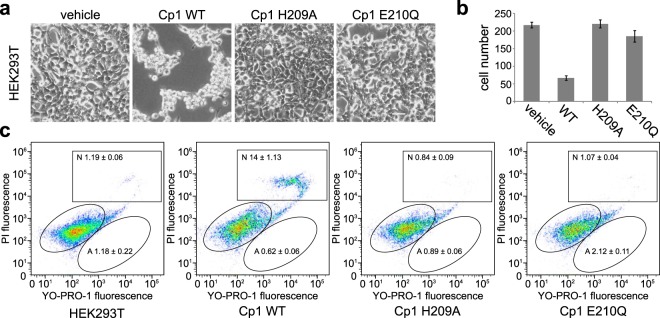
Expression of Cp1-LC in HEK 293T cell caused cell death, which is metalloprotease activity dependent. (**a**) HEK 293T cells were transfected with vehicle vector (pcDNA3.1(+)), Cp1-LC WT, and two mutants (H209A, E210Q) which abolished metalloprotease activity. Cells were observed and images were taken under inverted microscopy after 48 hrs. Cells transfected with Cp1-LC WT showed a dramatic cell death phenotype, becoming shrunken and detaching from the plate. Cells transfected with vehicle vector and protease activity-abolishing point mutants were not affected. (**b**) Cell numbers were counted in defined field of images taken in (**a)**. The number of cells transfected with CP1-LC WT were dramatically reduced compared to other treated cells. (**c**) Cell apoptosis assay was carried out with Chromatin Condenstion/Memberane Permeability/Dead cell Apoptosis kit. Transfected cells with vehicle vector, CP1-LC WT and mutant plasmids were analyzed by flow cytometry. Transfection with the CP1-LC WT plasmid increased the necrotic population of HEK 293 T cells, which is not observed in cells transfected with the vehicle vector or Cp1 point mutants.

As shown in Fig. 5a, the expression of wild-type Cp1-LC resulted in a cell death phenotype in HEK293T cells. These cells stopped proliferating and were visibly shrunken, eventually dying and detaching from culture plates. Cell counts after 48 hours revealed an almost 4-fold reduction in the number of cells (Fig. 5b). No cell death phenotype or significant reduction in cell number was observed in the H209A and E210Q mutants, confirming that the observed toxicity is likely metalloprotease-dependant. To further confirm the effect of Cp1-LC, we performed cell apoptosis assays by flow cytometry using Hoechst 33342, YO-PRO-1, and propidium iodide (Fig. 5c). In this assay, live cells, apoptotic cells (green), and dead cells (bright red and some green) are visualized by fluorescence. The percentage of necrotic death was much higher in cells transfected with Cp1-LC than in cells transfected with control plasmid, H209A, or E210Q mutants (greater than 10-fold increase). In contrast, the percentage of cells labeled as apoptotic death did not change appreciably. These results suggest that expression of Cp1-LC leads to necrotic death of cells, and that the cell death depends on the protease activity of Cp1-LC, although the specific target(s) of Cp1-LC remains unknown at this point.

## Discussion

Our survey of existing bacterial genomes reveals a diverse set of BoNT-related proteins outside of *Clostridium* such as those present in *Chryseobacterium* spp., a genus that includes pathogens of non-human hosts. Compared to the recently discovered BoNT in *Enterococcus faecium* (BoNT/En, ~29% overall sequence identity to BoNTs across full length sequence) and the BoNT homolog in *Weissella oryzae* (~20% overall identity to BoNTs), the *Chryseobacterium* BoNT-like toxins are more distantly related to BoNTs (~15% overall identity to BoNTs). This is supported by sequence and phylogenetic analysis of the light chain, which demonstrates that the homologs from *C*. *piperi* and other species cluster outside of the BoNT family (Fig. 2a). In addition, there are numerous other features present in the BoNT family that are lacking in *C*. *piperi*; specifically, the *C*. *piperi* toxins have weaker alignments to the translocation domain, lack the belt region, lack a detectable LamG-like H_CN_ domain present in the BoNT family, and lack the toxin accessory genes neighbouring the proteolytic toxin gene (including NTNH, and HA or P47/ORFX proteins). These trait differences suggest that, if BoNTs and *C*. *piperi* toxins do indeed share common ancestry, one of two scenarios have taken place: either the divergent BoNT homologs have lost some of these key BoNT features, or alternatively, these features emerged along with the BoNT lineage and may have differentiated BoNTs from their evolutionary relatives. Although one cannot distinguish between these two scenarios conclusively, we postulate that the latter scenario is more likely given the increased taxonomic and sequence diversity observed in the divergent BoNT-like toxin lineages.

The cytotoxic activity of *C*. *piperi* toxin in HEK293 cells, combined with the lack of protease activity against common BoNT substrates, suggests that the *C*. *piperi* toxin may have different targets in human cells. Given the degree of sequence and structural conservation observed between the protease domains of *C*. *piperi* toxins and BoNT-LC, it is tempting to speculate that *C*. *piperi* toxins may target different proteins with characteristics similar to SNAREs such as coiled-coil motifs. The future identification of the substrates targeted by *Chryseobacterium* toxin, *Weissella* toxin and others, combined with determination of their structure, will be important for not only illuminating the function and mechanism of these new toxins, but understanding the evolutionary novelties that occurred within the BoNT LC responsible for its gain of activity against neuronal SNAREs. Further, it will be important to explore the functionality of full-length Cp toxins and to determine whether they are expressed in their native host organism. Finally, if the protease domains of BoNT-related toxins have altered specificity to BoNT-LC, this may have significant biomedical and biotechnology applications through the engineering of BoNT-derived therapeutics that target different cell types.

## Methods

### Ethics Statement

Experiments were performed in accordance with the procedures approved by the Institutional Animal Care and Use Committee (IACUC) at Boston Children’s Hospital (protocol #3030). All experiments were performed in BSL-2 laboratory settings.

### Detection, comparison and analysis of bont-like genes

Sequences were retrieved using PSI-BLAST with default parameters (BLOSUM62 scoring matrix; expect threshold 10; gap open 11; extension 1) from the nr database on (March 26, 2017)^49^. Initial homologs were discovered by searching with BoNT/A1 (NCBI accession number ABS38337.1) with up to two rounds of PSI-BLAST. Then, in order to retrieve all possible sequences from each sequence family, different queries were used to search for specific BoNT homolog subfamilies (*Chryseobacterium*: WP_034681281.1, *Actinobacteria*: *Streptomyces* sp. NBRC 110027 GAO13068.1, fungal: *Metarhizium anisopliae* KFG81441.1) and reiterating to convergence. BoNT homologs identified this way were added to a set of known BoNT and NTNH proteins representing all known serotypes, including the recently discovered BoNT/F5A (KGO15617.1), BoNT/X (KGO12225.1), and BoNT/En (WP_086311652.1). Sets of M91 peptidases and diphtheria toxins were also retrieved via PSI-BLAST, with diphtheria toxin (PDB accession number 4AEO.1) and *E*. *coli* NleD (WP_069191536.1) as the original queries. These sets of M91 peptidases and diphtheria toxins were pruned to remove identical sequences using Jalview^50^.

All-by-all sequence pairwise alignments were generated with needle (of the EMBOSS package, v6.6.0.0^51^) with default parameters (gap open = 11, gap extend = 1, EBLOSUM62 scoring matrix). In Fig. 1, percent similarity was used over percent identity in order to allow divergent homologs to cluster more accurately. Principal coordinate analysis was performed in R on a distance matrix of pairwise similarity values using the default dist() and cmdscale() functions.

Domains were annotated with hmmscan (v3.1b2, available from http://hmmer.org/) against the Pfam database v31.0^52^ with an *E*-value cutoff of 1e-6. Annotations were subsequently confirmed by comparison to the Conserved Domain Database with relaxed cutoffs (v3.16)^53^, and alignment to BoNTs. Full domain annotations are available upon request. For Fig. 1, the BoNT homologs with the most BoNT-like annotations were depicted to facilitate comparison between categories.

### Comparison of proteases from BoNTs, BoNT-like proteins and M91 peptidases

All BoNT homologs possessing a putative peptidase domain (i.e., possessing an HExxH motif) were aligned with BoNT and M91 peptidases using Clustal-Omega with defaults v1.2.1^54^, manipulated and colored in Jalview^50^. Only regions corresponding to the peptidase domain boundaries were used, the positions of which were estimated based on alignment with domain boundaries of BoNT/A1 (PDB structure 3BTA). The same alignment procedure was used to identify the putative translocation region of BoNT homologs (Fig. 3). After identifying putative domains in BoNT homologs, the segments were combined and realigned.

A maximum likelihood phylogeny for BoNT, BoNT homolog and M91 peptidases was generated using RAxML (v8.2.4)^55^ with automatic model selection and 4 gamma-distributed rate categories (see simplified cladogram in Fig. 2, for the full tree see Fig. S3a). Bootstrap support was calculated using 1000 rapid bootstraps. The same alignment was used to infer a Bayesian phylogeny using MrBayes^56^ (with the ML-selected substitution model VT, 4 gamma-distributed rate categories, and 1,000,000 MCMC samplings; the consensus tree with 25% burn-in is depicted in Fig. S3b).

Pairwise global alignments were generated using BoNT/A1 (ABS38337.1) against the 220,362 metallopeptidase sequences available in the MEROPS database (retrieved Feb. 7, 2018) using needle from the EMBOSS package (v6.6.0.0)^51^. The alignment parameters were as follows: a gap open penalty of 11 and gap extension 1, with the BLOSUM62 substitution matrix. Raw alignment scores were averaged across peptidase families according to their MEROPS group, and visualized in R.

### Structural modelling of BoNT-like proteins and M91 peptidases

Structural templates were identified for Cp1 (*C*. *piperi*, accession WP_034687872.1) using the LOMETS meta-server^57^ on July 18, 2017. Templates (PDB IDs: 3BTA:A, 1XTG:A, 5BQN:A) were selected based on highest significant threading alignments (normalized Z-scores: 5.12-1.21, identity: 17–21%). Structural modelling and refinement was done through I-TASSER^58^, and the model with the lowest C-score was selected. For *E*. *coli* NleD, structural templates were identified through GeneSilico Metaservers (PDB IDs: 1Z7H:A, 1EPW:A, 3BWI:A, 3DEB:A, 3BON:A, 2QN0:A, 2A97:A, 3DDA:A, 1XTG:A, 1ZB7:A, 1F0L:A, 3FFZ:A, 1YVG:A, 2FPQ:A, 2G7K, 5BQN:A, 2NYY:A, 1T3C:A, 3V0A:A, 3FIE:A, 1RM8:A, 1E1H:A, 3VUO:A, 2A8A:A, 3D3X:A, 3DSE:A) were selected from COMA (score ≤ 5.4e-07, identity: 19%), HHblits (score: 100, identity: 13–20%), and HHsearch (score: 96.3, identity: 13–19%) on July 15, 2017. Structural modelling was carried out through PRIMO’s pipeline^59^. Identified template sequences were aligned to M91 with T-Coffee Expresso^60^, which uses 3D-Coffee to incorporate structural information during alignment. A total of 20 homology models were created with slow refinement based on the resulting alignment using MODELLER^61^. The model with the lowest DOPE Z-score was selected. Structural quality was assessed with Ramachandran plot analysis using PROCHECK^62^. Models were visualized using Chimera^63^.

### Re-sequencing and annotation of the *Chryseobacterium piperi* genome

Methods, materials, and platforms used in the sequencing and assembly of *C*. *piperi* are described in Wentz *et al*.^64^. The closed genome is accessible at the DDBJ/ENA/Genbank under the accession number CP023049. MiSeq and RS-II reads utilized in assembly are available at NCBI SRA under accessions SRX3229522, SRX3231351, SRX3231352. Figure 4 was generated using the program Circos^65^.

### HEK 293T cell transfection and cell number counting

HEK 293T cells were dispensed on 24-well plate at the density of 0.2 × 10^6^ cells/well. After 24 h, cells were transfected with 0.5 µg vehicle vector (pcDNA3.1(+)), Cp1-LC WT(1-398 aa), Cp1-LC H209A and Cp1-LC E210Q plasmids with PolyJet reagent. Pictures were taken 48 hours after transfection. Cell numbers were counted and combined from three different pictures.

### HEK 293T cell death assay

HEK 293T cells were dispensed on 60 mm dish at the density of 2.5 × 10^6^ cells/dish. Cells were transfected with 2.5 µg vehicle vector (pcDNA3.1(+)), Cp1-LC WT, Cp1-LC H209A and Cp1-LC E210Q plasmids by using 5 µl PolyJet. Cells were harvested 24 hrs after transfection and washed with cold phosphate-buffered saline (PBS). Cell density was adjusted to1 × 10^6^ cells/mL. Hoechst 33342, YO-PRO-1 and propidium iodide stock solution (1 µL Invitrogen) were added into 1 mL cell suspension. Cells were incubated on ice for 20–30 min. Stained cells were analyzed by flow cytometry (BD/Cytek FACSCalibur DxP 11). UV excitation was used for detection of 460 nm emission of Hoechst 33342 dye, 488 nm excitation was used for detection of the 530 nm emission of YO-PRO-1 dye, and 575 nm emission of propidium iodide. Cell populations separated into three groups: live cells showed low levels of blue fluorescence, apoptotic cells showed bright green and blue fluorescence, and necrotic cells showed bright red fluorescence.

### Cleavage of SNARE proteins by Cp1-LCs

HEK293T cells were dispensed on 24-well plate at the density of 0.3 × 10^6^ cells/well. 24 h later, cells in a single well were transfected with 0.5 µg vehicle vector (pcDNA3.1(+)), Cp1-LC WT(1-398 aa), Cp1-LC H209A, Cp1-LC E210Q, together with syntaxin 1, SNAP25, VAMP2 in pEGFP-C1 as indicated in Fig. S5. Cells were harvested 48 hours after transfection and lysed in RIPA buffer (50 mM Tris, 1% NP40, 150 mM NaCl, 0.5% sodium deoxycholate, 0.1% SDS, 400 ml per 10-cm dish) plus protease inhibitors. Cleavage assay was conducted by mixing cell lysates of vehicle vector, Cp1-LC WT, Cp1-LC H209A, Cp1-LC E210Q and GFP fused syntaxin 1, SNAP25, VAMP2 respectively and incubating the mixtures at 37 °C for 30 minutes. Samples were analyzed by immunoblot.

## Supplementary information

Supplementary Information

## Data Availability

All data reported in the paper is available through NCBI GenBank and upon request.
