# Supplementary material for: Bioinformatic discovery of a toxin family in Chryseobacterium piperi with sequence similarity to botulinum neurotoxins
Source: Sci Rep. 2019 Feb 7;9:1634. doi: 10.1038/s41598-018-37647-8 (PMC6367388; doi:10.1038/s41598-018-37647-8)
Supplement: Supplementary file 1 — Supplementary Information [file 41598_2018_37647_MOESM1_ESM.pdf]

Supplementary Information:

Bioinformatic discovery of a toxin family in *Chryseobacterium piperi* with sequence similarity to botulinum neurotoxins

Michael James Mansfield[1¶], *Travis Gwynn Wentz[2¶], Sicai Zhang[3], Elliot Jeon Lee[1], Min Dong[3*], Shashi Kant Sharma[2*] & Andrew Charles Doxey[1*]

[1]Department of Biology, University of Waterloo, 200 University Ave. West, Waterloo, Ontario, N2L 3G1, Canada.

[2]Center for Food Safety and Applied Nutrition, United States Food and Drug Administration, College Park, MD, 20740

[3]Department of Urology, Boston Children's Hospital, Department of Microbiology and Immunobiology and Department of Surgery, Harvard Medical School, Boston, MA 02115, USA

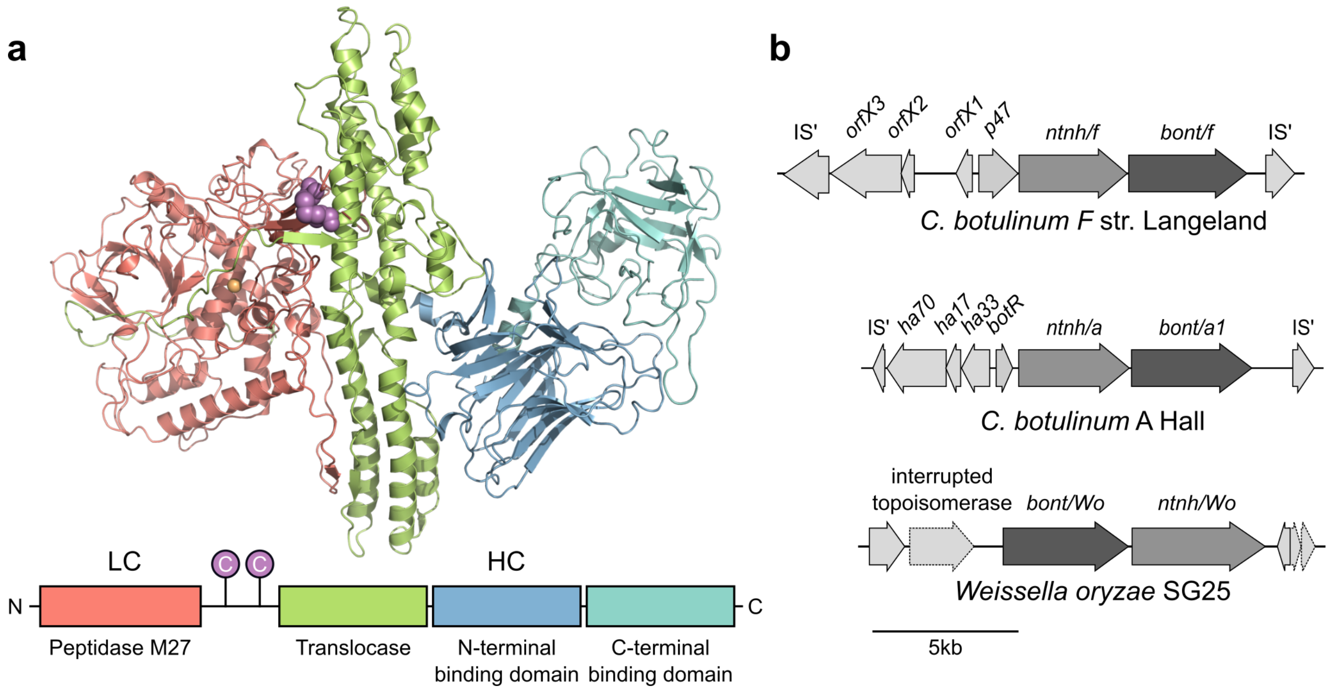

Figure S1. Botulinum neurotoxin (BoNT) protein and gene structure. (**a**) BoNTs are composed of four distinct structural domains. A single BoNT protein is cleaved into a smaller enzymatic component (the light chain, LC, which encodes a zinc endopeptidase) and larger binding and translocating component (the heavy chain, HC, which encodes the translocase domain and two binding domains). The HC is further subdivided into two subdomains (HC_N and H_{CC} which adopt a laminin G like beta-sandwich fold and a beta-trefoil fold, respectively). The light chain and heavy chain are linked by a disulfide bond. (**b**) BoNT genes are generally found in one of two main gene architectures. BoNT genes are always located next to a paralogous non-toxic non-hemagglutinin (NTNH) gene, but the two types are distinguished by their surrounding components, which consist of hemagglutinin (*ha*) or *orfX* genes. Currently, the only known example of altered synteny is in the unique *Weissella oryzae* BoNT homolog BoNT/Wo, where the *bont*/*ntnh* gene order has been reversed.

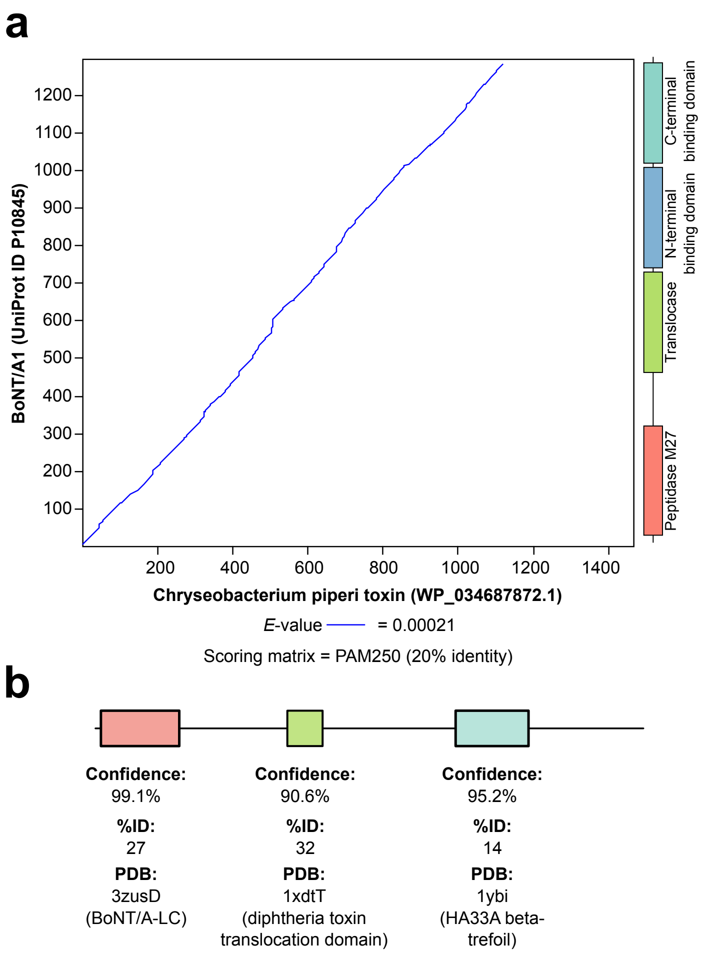

Figure S2. Pairwise local alignment and associated *E*-value between BoNT/A1 and *Chryseobacterium* toxin computed using lalign/plalign from the FASTA package. The alignment shown in (**a**) was generated using a PAM250 scoring matrix, which was selected since it models remote relationships with sequence identities of ~20%. The pairwise alignment spans multiple domains of BoNTs including the BoNT-LC, translocation domain and binding domain. (**b**) Domain structure predicted by Phyre version 2.0.

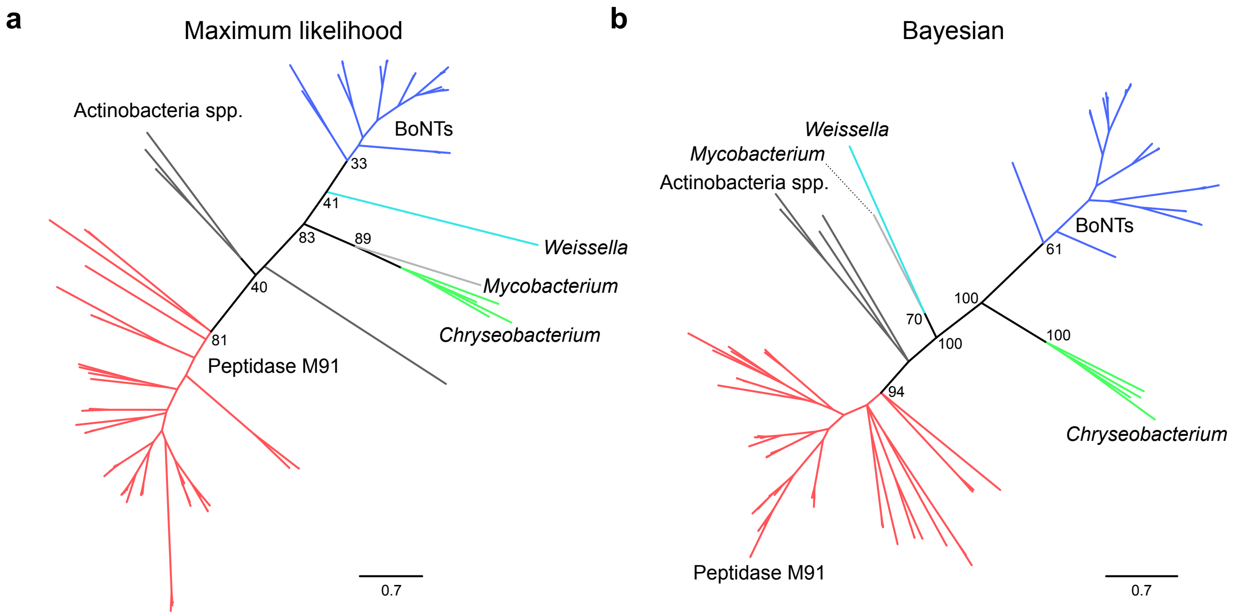

Figure S3. Phylogeny of BoNT, distant BoNT homologs and M91 peptidase domains. In both the maximum likelihood (**a**) and Bayesian (**b**) phylogenies, the T3SS effectors form a monophyletic group distinct from the BoNTs and BoNT homologs. Although the branch order differs between the two methods, the group comprised of BoNT homologs from *Weissella, Chryseobacterium*, and *Mycobacterium* form well-supported lineages in each tree. Within the BoNTs, each BoNT serotype clusters together, and in both trees the distant BoNT homologs found in the phylum Actinobacteria are the most distantly related. The ML phylogeny was generated using RAxML (v8.2.4), with 1000 rapid bootstraps, and the Bayesian tree with 1,000,000 MCMC generations using MrBayes (v3.2.4). Support values values are indicated at each node (bootstrap values for maximum likelihood and posterior probability percentage for Bayesian).

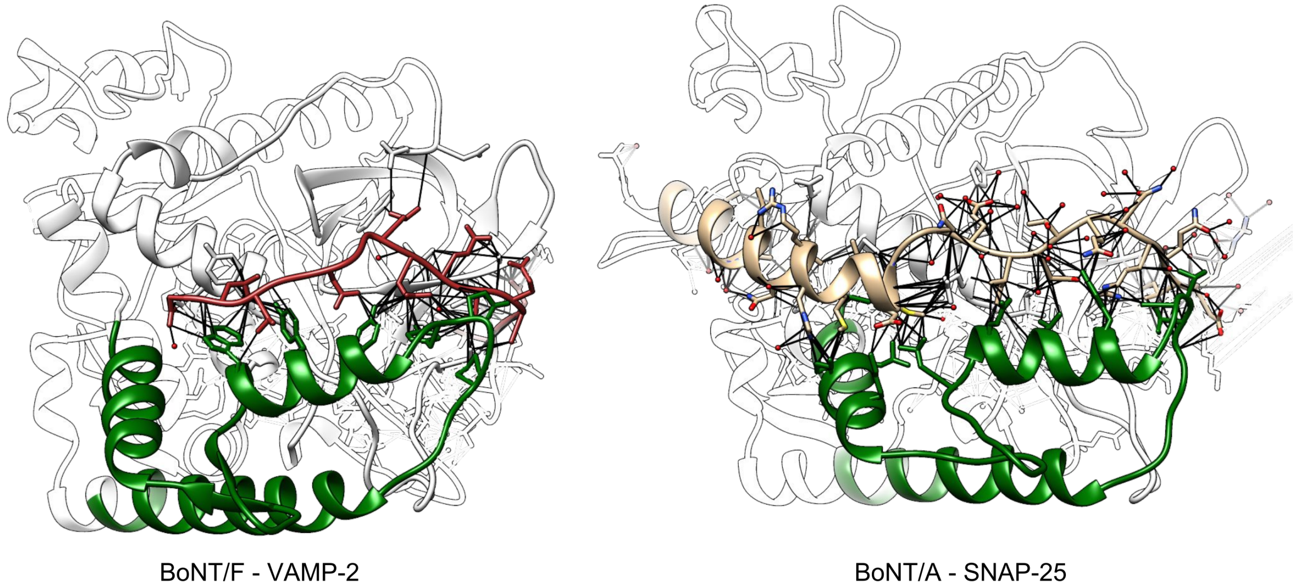

Figure S4. Structural visualization of BoNT-LC specific insertions. BoNT LC-specific insertions (dark green) share extensive contacts (black lines) with VAMP-2 (red) and SNAP25 (tan) in co-crystal complexes with BoNT/F (PDB ID: 3FIE) and BoNT/A (PDB ID: 1XTG), respectively.

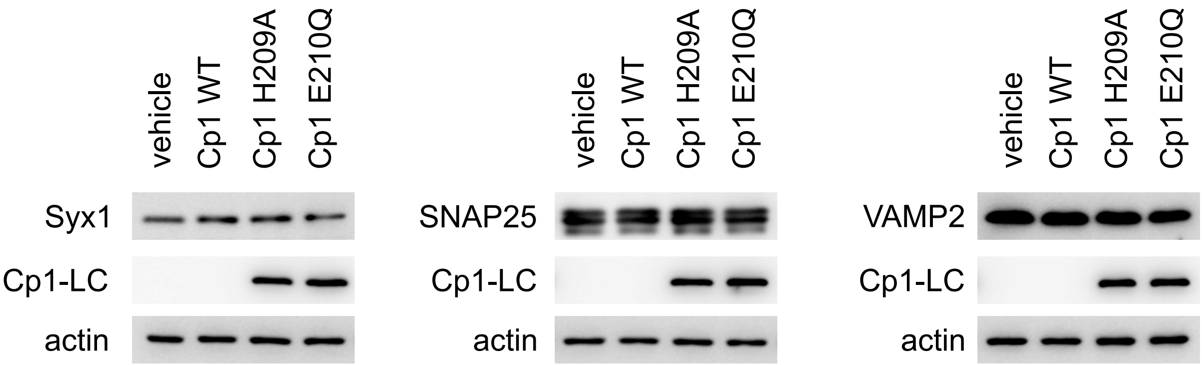

Figure S5. Cp1 does not cleave syntaxin 1, SNAP25, or VAMP2. FLAG-tagged Cp1-LC WT and mutants were expressed in HEK293T cells via transient transfection. GFP-tagged syntaxin 1 (Syx1), SNAP25, and VMP2 were expressed in HEK239 cells via transient transfection as well. Cells were harvested 48 h later after transfection and lysed in RIPA buffer. Cleavage of SNARE proteins was evaluated by mixing the cell lysates containing Syx1, SNAP25 or VAMP2 with the lysates containing Cp1 WT or mutants at 37 °C for 30 min. Samples were then analyzed by immunoblots. SNARE proteins were blotted with Syx1, SNAP25, and VAMP2 antibodies, respectively. Cp1-LC proteins were blotted with anti-FLAG antibody. Actin served as control for loading.

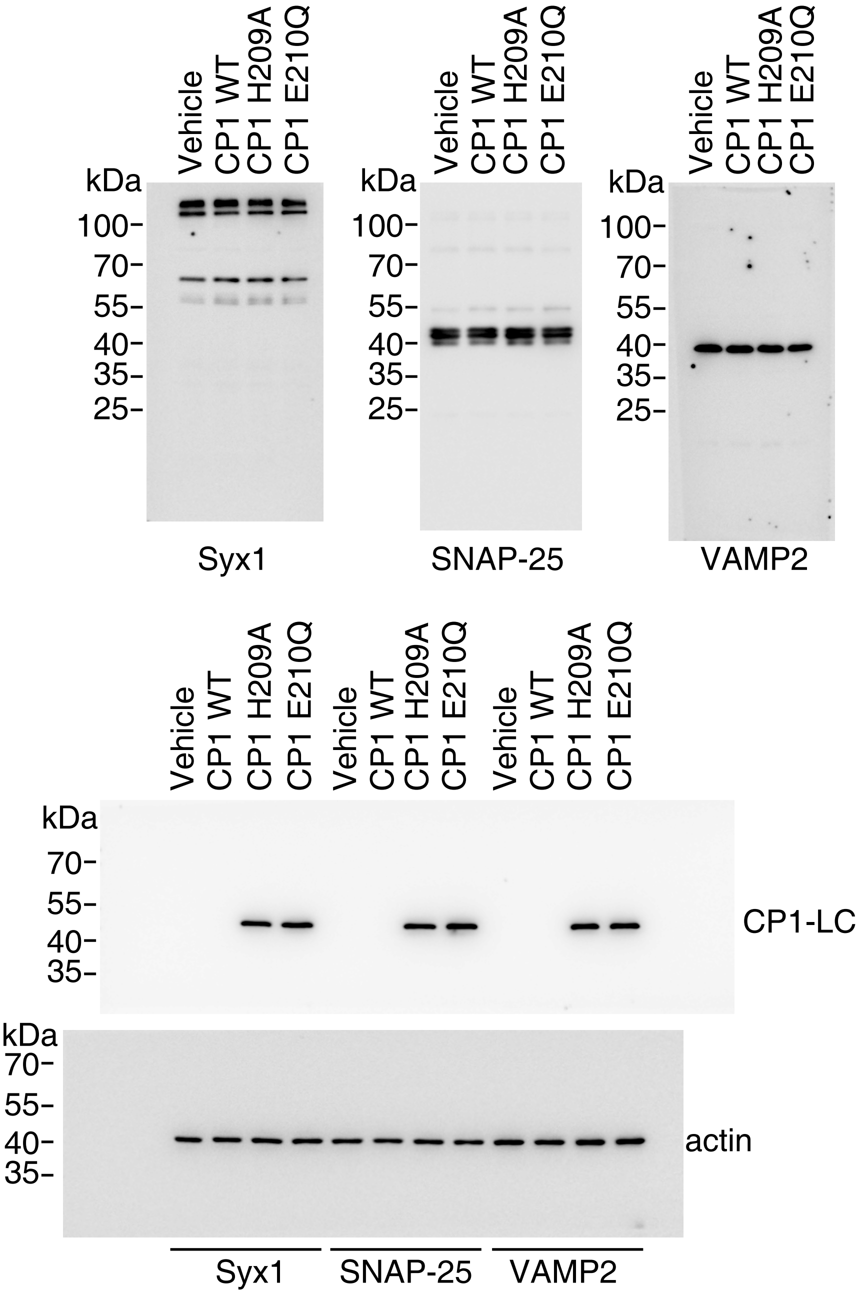

Figure S6. Uncropped images of gels from Fig. S5.

Table S1. Sequence identifiers, categories and protein lengths for each sequence used in this study. All sequences were retrieved from the NCBI nr database and are publicly available.

Accession	Species Name	Category	Length
WP_055473237.1	*Streptomyces pathocidini*	*Actinobacteria*	1066
WP_083906476.1	*Acaricomes phytoseiuli*	*Actinobacteria*	1370
SDT83331.1	*Streptomyces* sp. TLI 053	*Actinobacteria*	495
WP_058043469.1	*Streptomyces* sp. MBT76	*Actinobacteria*	512
EFL04418.1	*Streptomyces* sp. AA4	*Actinobacteria*	2761
WP_030364034.1	*Streptomyces roseoverticillatus*	*Actinobacteria*	519
SDS61334.1	*Streptomyces* sp. TLI 053	*Actinobacteria*	231
GAO13068.1	*Streptomyces* sp. NBRC 110027	*Actinobacteria*	841
BAF91946.1	*Clostridium botulinum* str. Osaka 05 BoNT B6	BoNT	1291
ABM73981.1	*Clostridium botulinum* BoNT E2	BoNT	1252
AFV91339.1	*Clostridium botulinum* str. CDC66177 BoNT E9	BoNT	1251
ACQ51417.1	*Clostridium botulinum* Ba4 str. 657 BoNT BvA4	BoNT	1296
KEI05265.1	*Clostridium botulinum* CD str. BKT2873 BoNT CD	BoNT	1291
AEN25581.1	*Clostridium botulinum* BoNT B7	BoNT	1291
ADA79573.1	*Clostridium botulinum* BoNT F5	BoNT	1277
ABM73977.1	*Clostridium botulinum* str. CDC 795 BoNT B3	BoNT	1291
ACA46990.1	*Clostridium botulinum* B1 str. Okra BoNT Ba4	BoNT	1291
CAA44558.1	*Clostridium botulinum* BoNT E1	BoNT	1252
BAP25804.1	*Clostridium botulinum* BoNT B2	BoNT	1291
CAA38175.1	*Clostridium botulinum* BoNT D	BoNT	1276
ADA79566.1	*Clostridium botulinum* BoNT F3	BoNT	1279
AER11392.1	*Clostridium botulinum* str. E134 BoNT E8	BoNT	1252
EEP52948.1	*Clostridium butyricum* E4 str. BoNT E BL5262 BoNT E4	BoNT	1252
ABS38337.1	*Clostridium botulinum* A str. Hall BoNT A1	BoNT	1296
EDT74844.1	*Clostridium butyricum* 5521 BoNT E	BoNT	1252
CBA17654.1	*Clostridium botulinum* BoNT CD	BoNT	1280
ABS41202.1	*Clostridium botulinum* F str. Langeland BoNT F1	BoNT	1278
ACD14195.1	*Clostridium botulinum* B str. Eklund 17B BoNT B4	BoNT	1291
ADU57954.1	*Clostridium botulinum* BoNT F6	BoNT	1275
ABM73985.1	*Clostridium botulinum* BoNT B2	BoNT	1291
ACT33194.1	*Clostridium botulinum* BoNT A5	BoNT	1296
EES49627.1	*Clostridium botulinum* E1 str. BoNT E Beluga BoNT E1	BoNT	1252
BAH84879.1	*Clostridium botulinum* BoNT DC	BoNT	1285
ACA57525.1	*Clostridium botulinum* A3 str. Loch Maree BoNT A3	BoNT	1292
EDS76240.1	*Clostridium botulinum* C str. Eklund BoNT C	BoNT	1280
CAA52275.1	*Clostridium botulinum* BoNT G	BoNT	1297
BAD90567.1	*Clostridium botulinum* BoNT C	BoNT	1291
BAB03522.1	*Clostridium butyricum* str. LCL 095 BoNT E5	BoNT	1251
ADA79579.1	Clostridium baratii BoNT F7	BoNT	1268
ACO83782.1	*Clostridium botulinum* A2 str. Kyoto BoNT A2	BoNT	1296
BAQ12790.1	*Clostridium botulinum* str 111 BoNT X	BoNT	1306
KGO12225.1	*Clostridium botulinum* BoNT H BoNT B	BoNT	1291
ADA79562.1	*Clostridium botulinum* BoNT F4	BoNT	1277
ADA79557.1	*Clostridium botulinum* BoNT F2	BoNT	1280
AER11391.1	*Clostridium botulinum* str. IBCA97 BoNT E7	BoNT	1252
CAM91125.1	*Clostridium botulinum* E str. K35 BoNT E6	BoNT	1252
KGO15617.1	*Clostridium botulinum* BoNT H BoNT Fa	BoNT	1288

KEH96501.1	*Clostridium botulinum* D str. 16868 BoNT D	BoNT	1287
ACQ51206.1	*Clostridium botulinum* Ba4 str. 657 BoNT BvBb5	BoNT	1291
ACD53549.1	*Clostridium botulinum* E3 str. Alaska E43 BoNT E3	BoNT	1252
WP_034687877.1	*Chryseobacterium piperi*	*Chryseobacterium*	388
KFF17709.1	*Chryseobacterium piperi* partial	*Chryseobacterium*	2112
WP_034687879.1	*Chryseobacterium piperi*	*Chryseobacterium*	695
WP_034681279.1	*Chryseobacterium piperi*	*Chryseobacterium*	1747
WP_034687193.1	*Chryseobacterium piperi*	*Chryseobacterium*	1496
WP_034687872.1	*Chryseobacterium piperi*	*Chryseobacterium*	1467
WP_034681281.1	*Chryseobacterium piperi*	*Chryseobacterium*	1617
WP_034687874.1	*Chryseobacterium piperi*	*Chryseobacterium*	1116
NZ_BAGZ01000024:39931_43386	*Austwickia chelonae*	Diphtheria	260
WP_040322835.1	*Austwickia chelonae*	Diphtheria	274
WP_073156187.1	*Seinonella peptoniphila*	Diphtheria	606
ABU25232.1	Corynephage beta	Diphtheria	563
4AE0.1	*Corynebacterium diphtheria*	Diphtheria	535
WP_071569945.1	*Corynebacterium diphtheriae*	Diphtheria	560
BAG06869.1	*Corynebacterium ulcerans*	Diphtheria	560
WP_029975703.1	*Corynebacterium ulcerans*	Diphtheria	560
WP_038617330.1	*Corynebacterium ulcerans*	Diphtheria	560
WP_044032678.1	*Corynebacterium ulcerans*	Diphtheria	560
AAV70486.1	*Corynebacterium diphtheriae*	Diphtheria	536
AND74674.1	*Corynebacterium diphtheriae* bv. mitis	Diphtheria	560
AGT63319.1	*Corynebacterium ulcerans*	Diphtheria	296
WP_014654963.1	*Corynebacterium pseudotuberculosis*	Diphtheria	560
BAB03348.1	Corynephage beta	Diphtheria	535
AMP42519.1	*Corynebacterium diphtheriae*	Diphtheria	560
AMP42520.1	*Corynebacterium diphtheriae*	Diphtheria	560
WP_014835773.1	*Corynebacterium ulcerans*	Diphtheria	560
AAW22870.1	*Corynebacterium ulcerans*	Diphtheria	560
P00588.2	*Corynebacterium diphtheria*	Diphtheria	567
WP_054467370.1	*Corynebacterium ulcerans*	Diphtheria	560
WP_003850266.1	*Corynebacterium diphtheria*	Diphtheria	560
KHN96101.1	*Metarhizium album* ARSEF 1941	Fungal	730
XP_018137534.1	*Pochonia chlamydosporia* 170	Fungal	874
XP_007815863.1	*Metarhizium acridum* CQMa 102	Fungal	905
KID82327.1	*Metarhizium guizhouense* ARSEF 977	Fungal	994
KID81771.1	*Metarhizium guizhouense* ARSEF 977	Fungal	734
KFG79709.1	*Metarhizium anisopliae*	Fungal	704
XP_007806665.1	*Metarhizium acridum* CQMa 102	Fungal	795
KJZ74539.1	*Hirsutella minnesotensis* 3608	Fungal	951
KHN97968.1	*Metarhizium album* ARSEF 1941	Fungal	707
XP_007825170.1	*Metarhizium robertsii* ARSEF 23	Fungal	1082
KOM18317.1	*Ophiocordyceps unilateralis*	Fungal	723
KJK83643.1	*Metarhizium anisopliae* BRIP 53293	Fungal	1048
KFG84249.1	*Metarhizium anisopliae*	Fungal	909
KID84774.1	*Metarhizium guizhouense* ARSEF 977	Fungal	1081
KID84474.1	*Metarhizium guizhouense* ARSEF 977	Fungal	290
XP_018138178.1	*Pochonia chlamydosporia* 170	Fungal	1069
KFG84771.1	*Metarhizium anisopliae*	Fungal	994
XP_014548865.1	*Metarhizium brunneum* ARSEF 3297	Fungal	908

EQL01877.1	*Ophiocordyceps sinensis* CO18	Fungal	615
XP_014541277.1	*Metarhizium brunneum* ARSEF 3297	Fungal	1082
KOM20214.1	*Ophiocordyceps unilateralis*	Fungal	798
XP_008600141.1	*Beauveria bassiana* ARSEF 2860	Fungal	172
XP_006673829.1	*Cordyceps militaris* CM01	Fungal	990
KOM18061.1	*Ophiocordyceps unilateralis*	Fungal	1006
KYK53973.1	*Drechmeria coniospora*	Fungal	753
OAR01471.1	*Cordyceps confragosa*	Fungal	901
XP_006672770.1	*Cordyceps militaris* CM01	Fungal	1046
KOM19981.1	*Ophiocordyceps unilateralis*	Fungal	676
XP_018701747.1	*Isaria fumosorosea* ARSEF 2679	Fungal	966
KJZ72439.1	*Hirsutella minnesotensis* 3608	Fungal	732
KJK78274.1	*Metarhizium anisopliae* BRIP 53293	Fungal	994
KOM17722.1	*Ophiocordyceps unilateralis*	Fungal	692
KID81025.1	*Metarhizium guizhouense* ARSEF 977	Fungal	1026
KFG78556.1	*Metarhizium anisopliae*	Fungal	1082
KID81342.1	*Metarhizium guizhouense* ARSEF 977	Fungal	1051
KHN96509.1	*Metarhizium album* ARSEF 1941	Fungal	799
XP_007813862.1	*Metarhizium acridum* CQMa 102	Fungal	731
KHN94110.1	*Metarhizium album* ARSEF 1941	Fungal	850
OAA45953.1	*Cordyceps brongniartii* RCEF 3172	Fungal	896
XP_014576605.1	*Metarhizium majus* ARSEF 297	Fungal	707
XP_007821069.1	*Metarhizium robertsii* ARSEF 23	Fungal	680
KOM19459.1	*Ophiocordyceps unilateralis*	Fungal	1134
KJK74733.1	*Metarhizium anisopliae* BRIP 53293	Fungal	777
XP_008597266.1	*Beauveria bassiana* ARSEF 2860	Fungal	905
XP_007823153.1	*Metarhizium robertsii* ARSEF 23	Fungal	994
KHN96007.1	*Metarhizium album* ARSEF 1941	Fungal	182
XP_014574393.1	*Metarhizium majus* ARSEF 297	Fungal	1052
KOM22197.1	*Ophiocordyceps unilateralis*	Fungal	872
KFG79783.1	*Metarhizium anisopliae*	Fungal	790
KFG84514.1	*Metarhizium anisopliae*	Fungal	680
OAA46382.1	*Metarhizium rileyi* RCEF 4871	Fungal	1144
KGQ06896.1	*Beauveria bassiana* D1	Fungal	958
XP_007815132.1	*Metarhizium acridum* CQMa 102	Fungal	700
KZZ89413.1	*Aschersonia aleyrodis* RCEF 2490	Fungal	775
OAA38271.1	*Cordyceps brongniartii* RCEF 3172	Fungal	957
KFG81441.1	*Metarhizium anisopliae*	Fungal	961
KID85357.1	*Metarhizium guizhouense* ARSEF 977	Fungal	908
KOM22373.1	*Ophiocordyceps unilateralis*	Fungal	1051
ODA78204.1	*Drechmeria coniospora*	Fungal	795
KJK91804.1	*Metarhizium anisopliae* BRIP 53284	Fungal	1041
XP_008593942.1	*Beauveria bassiana* ARSEF 2860	Fungal	892
XP_018701218.1	*Isaria fumosorosea* ARSEF 2679	Fungal	864
KJK74458.1	*Metarhizium anisopliae* BRIP 53293	Fungal	300
KJZ74925.1	*Hirsutella minnesotensis* 3608	Fungal	779
KOM17891.1	*Ophiocordyceps unilateralis*	Fungal	800
KGQ09460.1	*Beauveria bassiana* D1	Fungal	892
KJZ75232.1	*Hirsutella minnesotensis* 3608	Fungal	967
OAA33269.1	*Aschersonia aleyrodis* RCEF 2490	Fungal	776
KOM19891.1	*Ophiocordyceps unilateralis*	Fungal	684

KJK77338.1	*Metarhizium anisopliae* BRIP 53293	Fungal	909
XP_018141808.1	*Pochonia chlamydosporia* 170	Fungal	836
KJZ77068.1	*Hirsutella minnesotensis* 3608	Fungal	651
KZZ93685.1	*Aschersonia aleyrodis* RCEF 2490	Fungal	882
KOM20843.1	*Ophiocordyceps unilateralis*	Fungal	667
EQL03202.1	*Ophiocordyceps sinensis* CO18	Fungal	707
XP_018702425.1	*Isaria fumosorosea* ARSEF 2679	Fungal	970
OAA33955.1	*Cordyceps brongniartii* RCEF 3172	Fungal	984
KZZ97314.1	*Aschersonia aleyrodis* RCEF 2490	Fungal	1053
XP_014543958.1	*Metarhizium brunneum* ARSEF 3297	Fungal	1055
KOM18203.1	*Ophiocordyceps unilateralis*	Fungal	759
OAA38924.1	*Cordyceps brongniartii* RCEF 3172	Fungal	835
KID83117.1	*Metarhizium guizhouense* ARSEF 977	Fungal	802
KHN95172.1	*Metarhizium album* ARSEF 1941	Fungal	686
KZZ88788.1	*Aschersonia aleyrodis* RCEF 2490	Fungal	634
KHO00998.1	*Metarhizium album* ARSEF 1941	Fungal	846
XP_006671066.1	*Cordyceps militaris* CM01	Fungal	1164
OAA70995.1	*Cordyceps confragosa* RCEF 1005	Fungal	953
KOM19467.1	*Ophiocordyceps unilateralis*	Fungal	620
EQL04075.1	*Ophiocordyceps sinensis* CO18	Fungal	965
KFG86514.1	*Metarhizium anisopliae*	Fungal	1048
KHO02117.1	*Metarhizium album* ARSEF 1941	Fungal	907
XP_007825853.1	*Metarhizium robertsii* ARSEF 23	Fungal	908
KJK76584.1	*Metarhizium anisopliae* BRIP 53293	Fungal	782
XP_014544711.1	*Metarhizium brunneum* ARSEF 3297	Fungal	680
KID86267.1	*Metarhizium guizhouense* ARSEF 977	Fungal	467
KID82428.1	*Metarhizium guizhouense* ARSEF 977	Fungal	644
KID61384.1	*Metarhizium anisopliae* ARSEF 549	Fungal	782
EXU95574.1	*Metarhizium robertsii*	Fungal	631
ODA76424.1	*Drechmeria coniospora*	Fungal	969
XP_014581715.1	*Metarhizium majus* ARSEF 297	Fungal	903
KZZ92552.1	*Aschersonia aleyrodis* RCEF 2490	Fungal	1024
KJK78185.1	*Metarhizium anisopliae* BRIP 53293	Fungal	1082
KJZ71660.1	*Hirsutella minnesotensis* 3608	Fungal	986
KID82801.1	*Metarhizium guizhouense* ARSEF 977	Fungal	819
KYK58955.1	*Drechmeria coniospora*	Fungal	969
KID82824.1	*Metarhizium guizhouense* ARSEF 977	Fungal	463
XP_007824532.1	*Metarhizium robertsii* ARSEF 23	Fungal	704
KJK84451.1	*Metarhizium anisopliae* BRIP 53293	Fungal	680
KHN94021.1	*Metarhizium album* ARSEF 1941	Fungal	673
XP_018700026.1	*Isaria fumosorosea* ARSEF 2679	Fungal	830
KGQ03580.1	*Beauveria bassiana* D1	Fungal	905
XP_014540776.1	*Metarhizium brunneum* ARSEF 3297	Fungal	994
KOM19284.1	*Ophiocordyceps unilateralis*	Fungal	1163
XP_014539926.1	*Metarhizium brunneum* ARSEF 3297	Fungal	790
OAA52226.1	*Cordyceps brongniartii* RCEF 3172	Fungal	945
KJK74970.1	*Metarhizium anisopliae* BRIP 53293	Fungal	704
KJK83642.1	*Metarhizium anisopliae* BRIP 53293	Fungal	305
XP_011411449.1	*Metarhizium robertsii* ARSEF 23	Fungal	827
KOM17955.1	*Ophiocordyceps unilateralis*	Fungal	960
XP_014576580.1	*Metarhizium majus* ARSEF 297	Fungal	759

ODA80573.1	*Drechmeria coniospora*	Fungal	857
XP_008599538.1	*Beauveria bassiana* ARSEF 2860	Fungal	219
KGQ05150.1	*Beauveria bassiana* D1	Fungal	458
KJZ71838.1	*Hirsutella minnesotensis* 3608	Fungal	756
KOM19347.1	*Ophiocordyceps unilateralis*	Fungal	1135
XP_014575725.1	*Metarhizium majus* ARSEF 297	Fungal	704
KYK56264.1	*Drechmeria coniospora*	Fungal	912
KJZ74384.1	*Hirsutella minnesotensis* 3608	Fungal	788
XP_008602550.1	*Beauveria bassiana* ARSEF 2860	Fungal	836
OAA52037.1	*Metarhizium rileyi* RCEF 4871	Fungal	711
XP_006674368.1	*Cordyceps militaris* CM01	Fungal	1035
XP_014574395.1	*Metarhizium majus* ARSEF 297	Fungal	637
EQL00592.1	*Ophiocordyceps sinensis* CO18	Fungal	940
XP_007816336.1	*Metarhizium robertsii* ARSEF 23	Fungal	1052
XP_008601493.1	*Beauveria bassiana* ARSEF 2860	Fungal	970
OAA75559.1	*Cordyceps confragosa* RCEF 1005	Fungal	931
KJZ70085.1	*Hirsutella minnesotensis* 3608	Fungal	471
OAA39888.1	*Cordyceps brongniartii* RCEF 3172	Fungal	902
KJZ78148.1	*Hirsutella minnesotensis* 3608	Fungal	775
WP_011267300.1	*Pseudomonas syringae*	M91	198
CUV34250.1	*Ralstonia solanacearum*	M91	245
AEX33777.1	*Xanthomonas arboricola* pv. pruni	M91	146
WP_077142976.1	*Pseudomonas syringae*	M91	215
WP_061231638.1	*Leptospira noguchii*	M91	334
WP_074375462.1	*Xanthomonas translucens*	M91	204
GAE50625.1	*Xanthomonas arboricola* pv. pruni str. MAFF 311562	M91	230
ADX47300.1	*Acidovorax avenae* subsp. avenae ATCC 19860	M91	212
WP_026053328.1	*Leptospira santarosai*	M91	245
WP_069342860.1	*Pandoraea* sp. ISTKB	M91	222
WP_074052393.1	*Xanthomonas vesicatoria*	M91	213
WP_071011456.1	*Ralstonia solanacearum*	M91	219
WP_024689564.1	*Pseudomonas syringae* group	M91	212
CAP52016.1	*Xanthomonas campestris* pv. campestris	M91	249
CTP91498.1	*Xanthomonas translucens* pv. poae	M91	221
WP_020738886.1	*Sorangium cellulosum*	M91	260
WP_004471278.1	*Leptospira santarosai*	M91	361
WP_057176762.1	*Paraburkholderia caribensis*	M91	206
AMV47536.1	*Paraburkholderia caribensis*	M91	284
AEG71857.1	*Ralstonia solanacearum* Po82	M91	225
WP_074812007.1	*Pseudomonas syringae*	M91	230
WP_064048191.1	*Ralstonia solanacearum*	M91	219
SEI45716.1	*Pseudomonas* sp. NFR16	M91	234
AIL29259.1	*Pseudomonas syringae* pv. actinidiae	M91	201
WP_019994798.1	*Aureimonas ureilytica*	M91	179
WP_075251160.1	*Xanthomonas oryzae*	M91	168
WP_006453000.1	*Xanthomonas gardneri*	M91	218
WP_064297220.1	*Ralstonia solanacearum*	M91	221
AIE45643.1	*Acidovorax citrulli*	M91	197
WP_035542658.1	Burkholderia sp. UYPR1.413	M91	238
WP_006073522.1	*Vibrio*	M91	214
WP_039558791.1	*Ralstonia solanacearum*	M91	186

WP_011409781.1	*Xanthomonas oryzae*	M91	155
WP_036949404.1	*Providencia alcalifaciens*	M91	240
WP_063885037.1	*Pseudomonas syringae*	M91	183
KPY01823.1	*Pseudomonas* amygdali pv. mori	M91	212
KPY92388.1	*Pseudomonas syringae* pv. tomato	M91	236
WP_019702312.1	*Acidovorax*	M91	213
WP_069191536.1	*Escherichia coli*	M91	231
GAE54985.1	*Xanthomonas arboricola* pv. pruni MAFF 301420	M91	219
WP_043897868.1	*Ralstonia solanacearum*	M91	174
WP_051781314.1	*Janthinobacterium agaricidamnosum*	M91	223
WP_076037893.1	*Xanthomonas campestris*	M91	207
WP_004771984.1	*Leptospira kirschneri*	M91	283
WP_017115174.1	*Xanthomonas vasicola*	M91	206
WP_016971449.1	*Pseudomonas tolaasii*	M91	184
WP_074686786.1	*Acidovorax citrulli*	M91	216
KFA31217.1	*Xanthomonas vasicola* pv. vasculorum NCPPB 1326	M91	202
KPY56004.1	*Pseudomonas* amygdali pv. sesami	M91	226
KPW50752.1	*Pseudomonas syringae* pv. berberidis	M91	221
CUV20876.1	*Ralstonia solanacearum*	M91	234
WP_075241300.1	*Xanthomonas oryzae*	M91	171
WP_071615581.1	*Ralstonia solanacearum*	M91	218
WP_061944074.1	*Collimonas pratensis*	M91	265
AMP07033.1	*Collimonas pratensis*	M91	304
KPX28235.1	*Pseudomonas* coronafaciens pv. garcae	M91	159
WP_075242222.1	*Xanthomonas oryzae*	M91	167
KTB84937.1	*Pseudomonas syringae* pv. syringae PD2774	M91	196
WP_011003174.1	*Ralstonia solanacearum*	M91	217
WP_071895715.1	*Ralstonia solanacearum*	M91	183
WP_070931164.1	*Mycobacterium chelonae*	*Mycobacterium*	990
WP_070931163.1	*Mycobacterium chelonae*	*Mycobacterium*	391
EES49602.1	*Clostridium botulinum* E1 str. BoNT E Beluga NTNH E1	NTNH	1163
ACD14165.1	*Clostridium botulinum* B str. Eklund 17B NTNH B4	NTNH	1196
KGO15578.1	*Clostridium botulinum* BoNT H NTNH A	NTNH	1164
ACD52603.1	*Clostridium botulinum* E3 str. Alaska E43 NTNH E3	NTNH	1163
ACA57431.1	*Clostridium botulinum* A3 str. Loch Maree NTNH A3	NTNH	1159
BAQ12789.1	*Clostridium botulinum* str 111 NTNH X	NTNH	1174
CAA61228.1	*Clostridium botulinum* NTNH G	NTNH	1198
BAF91945.1	*Clostridium botulinum* str. Osaka 05 NTNH B6	NTNH	1197
BAP25803.1	*Clostridium botulinum* str. Prevot 25 NTNH B2	NTNH	1197
KEH96500.1	*Clostridium botulinum* D str. 16868 NTNH D	NTNH	1196
KGO12234.1	*Clostridium botulinum* BoNT H NTNH B	NTNH	1197
EDS76246.1	*Clostridium botulinum* C str. Eklund NTNH C	NTNH	1196
EEP54802.1	*Clostridium butyricum* E4 str. BoNT E BL5262 NTNH E4	NTNH	1163
ACQ51342.1	*Clostridium botulinum* Ba4 str. 657 NTNH BivA4	NTNH	1159
CAM91124.1	*Clostridium botulinum* E NTNH E6	NTNH	1163
EDT74767.1	*Clostridium butyricum* 5521 NTNH E	NTNH	1163
KEI05264.1	*Clostridium botulinum* CD str. BKT2873 NTNH CD	NTNH	1196
ACT33193.1	*Clostridium botulinum* NTNH A5	NTNH	1193
ABS37375.1	*Clostridium botulinum* A str. Hall NTNH A1	NTNH	1193
ACA47084.1	*Clostridium botulinum* B1 str. Okra NTNH B1	NTNH	1197
ACO85717.1	*Clostridium botulinum* A2 str. Kyoto NTNH A2	NTNH	1159

ADU57953.1	*Clostridium botulinum* NTNH F6	NTNH	1165
ABS40335.1	*Clostridium botulinum* F str. Langeland NTNH F1	NTNH	1163
AGR53839.1	*Clostridium baratii* NTNH F7	NTNH	1162
ACQ51274.1	*Clostridium botulinum* Ba4 str. 657 NTNH BvB	NTNH	1197
AAO37454.1	*Clostridium tetani* E88 TeNT tetani	TeNT	1315
WP_027699549.1	*Weissella oryzae* SG25 WoNT	*Weissella*	1296
WP_027699548.1	*Weissella oryzae* SG25 WoNTNH	*Weissella*	1437

Table S2. Gene annotations for toxin gene clusters in *Chryseobacterium piperi*.

Accession	Clu-ster	Type/Name	Annotation	DNA Homology Inference	Protein Homology Inference (BLASTP Top 100)	General Notes
CJF12_06270	1	DS Flank	Fatty Acid Hydroxylase			
CJF12_06275	1	DS Flank	Short-Chain Dehydrogenase	Broad distribution in Chryseobacterium	Full length hit, <1e-131, Genera: Chryseobacterium, Pedobacter, Flavobacterium, …	
CJF12_06280	1	DS Flank	Hypothetical Protein (pseudo)	Broad distribution in Chryseobacterium	Full length hit, <4e-16, Genera: Chryseobacterium, Elizabethkingia, Cruoricaptor …	Peptidase M15 domain protein, Endolysin, Phage-like protein, Van-Y like
CJF12_06285	1	DS Flank	Transposase (IS21)	Broad distribution in Chryseobacterium	Full length hit, <1e-161, Genera: Chryseobacterium, Elizabethkingia, Flavobacterium, …	IS21
CJF12_06290	1	DS Flank	ATP-Binding Protein (IS21 Transposase)	Broad distribution in Chryseobacterium	Full length hit, <1e-94, Genera: Chryseobacterium, Elizabethkingia, Flavobacterium, …	IS21 AG
CJF12_06295	1	DS Flank	BoNT_GC1_4			
CJF12_06300	1	DS Flank	Hypothetical Protein	Broad distribution in Chryseobacterium	Full length hit,<7e-25, Genera: Chryseobacterium, Elizabethkingia, Cruoricaptor, …	Partial Domain Match D-alanyl-D-alanine carboxypeptidase
CJF12_06305	1	BoNT-Like	BoNT_GC1_3			
CJF12_06315	1	BoNT-Like	BoNT_GC1_3			
CJF12_06320	1	NTNH-Analog	NTNH_GC1_2			
CJF12_06335	1	BoNT-Like	BoNT_GC1_2			CP1 Toxin
CJF12_06345	1	NTNH-Analog	NTNH_GC1_1			
CJF12_06355	1	BoNT-Like	BoNT_GC1_1			
CJF12_06360	1	US Flank	Response Regulator	Broad distribution in Chryseobacterium	Full length hit, <1e-47, Genera: Chryseobacterium, Sphingobacterium	
CJF12_06365	1	US Flank	Chemotaxis Protein CheB	Broad distribution in Chryseobacterium	Full length hit, <2e-60, Genera: Chryseobacterium, Sphingobacterium, Pedobacter, …	
CJF12_06370	1	US Flank	Chemotaxis Protein CheR	Broad distribution in Chryseobacterium	Full length hit, <2e-136, Genera: Chryseobacterium, Sphingobacterium, Pedobacter, …	
CJF12_06375	1	US Flank	Response Regulator	Broad distribution in Chryseobacterium	Full length hit, <2e-44, Genera: Chryseobacterium, Sphingobacterium, Flavobacterium …	
CJF12_06380	1	US Flank	Histidine Kinase	Broad distribution in Chryseobacterium	Full length hit, <2e-44, Genera: Chryseobacterium, Sphingobacterium, Flavobacterium …	
CJF12_14515	2	Alcohol Dehydrogenase	Broad distribution in Chryseobacterium	Full length hit, <1e-127, Genera: Chryseobacterium, Chitinophaga, Flavobacterium, …		
CJF12_14520	2	Hypothetical Protein	Broad distribution in Chryseobacterium	Full length hit, = 0.0, Genus: Chryseobacterium	Annotation: Pos Tetraricopeptide repeat-containing protein	
CJF12_14525	2	IS1595 Family Transposase ISChpi1	Nucleotide homology to Elizabethkingia, Draconibacterium,	Full length hit, <2e-87, Broad Bacteroidetes distribution	IS1595, similar to CJF12_14620	

			Chryseobacterium, Myroides, …		
CJF12_14530	2	Hypothetical Protein	Low homology region	Low homology protein	
CJF12_14535	2	Hypothetical Protein	Low homology region	Full length hit, 2.6e-30, uncultured Bacteroidetes; partial C-term hits, <8e-20, Broad Bacteroidetes distribution	Annotation: Pos TMF family protein domain in hit Spirosoma luteum
CJF12_14540	2	Hypothetical Protein	Low homology region	Hits (6e-18 to 9e-06) to N-terminus of Glycerophosphodiester phosphordiesterase domain proteins.	Annotation: Pos Glycerophosphodiester phosphordiesterase domain of Agrobacterium tumefaciens and similar proteins.
CJF12_14545	2		N		
CJF12_14550	2		N		
CJF12_14555	2	IS1982 Family Transposase	Nucleotide homology to Genera: Prevotella, Clostridium, Barnesiella, …	Degraded, Broad Bacteroidetes distribution	
CJF12_14560	2	Alpha/Beta Hydrolase	Nucleotide homology to Mycobacterium chelonae CCUG 47445; Genera: Debaryomyces, Dickeya, Candida, Agarobacterium, Mycobacterium, Rhodococcus	Full length hit, <2e-97, Genera: Chryseobacterium, Mycobacterium, Rhodococcus, Variovorax, Actinomadura, Nocardia, Chintinophaga, Pararhizobium, Ensifer, *Pseudomonas*, …	
CJF12_14565	2	tRNA-Leu	Broad distribution in Chryseobacterium, Elizabethingia, Riemerella	N/A	tRNA
CJF12_14570	2	tRNA-Gly	Broad distribution in Chryseobacterium	N/A	tRNA
CJF12_14575	2	tRNA-Leu	Broad distribution in Chryseobacterium	N/A	tRNA
CJF12_14580	2	tRNA-Leu	Broad distribution in Chryseobacterium	N/A	tRNA
CJF12_14585	2	tRNA-Gly	Broad distribution in Chryseobacterium	N/A	tRNA
CJF12_14590	2	tRNA-Leu	Broad distribution in Chryseobacterium	N/A	tRNA
CJF12_14595	2	DUF3127 Domain-Containing Protein	Broad distribution in Chryseobacterium	Full length hit, <6e-51, Genera: Chryseobacterium, Flavobacterium, Riemerella	
CJF12_14600	2	Leucyl/Phenylalanyl-tRNA-Protein Transferase	Broad distribution in Chryseobacterium	Full length hit, <3e-107, Genus: Chryseobacterium	
CJF12_14605	2	EamA/RhaT Family Transporter	Broad distribution in Chryseobacterium	Full length hit, <2e-142, Genus: Chryseobacterium	*CJF12_14565 through CJF12_14605 are inverted
CJF12_14610	2	Ankyrin Repeat Domain Containing Protein	Limited Distribution within Chryseobacterium, Elizabethkingia, Sphingobacterium	Full length hit, <1e-37, Genera: Chryseobacterium, Elizabethkingia, Empedobacter Flavobacterium, …	

CJF12_14615_2	Catalase	Limited Distribution within Chryseobacterium, Elizabethkingia, Sphingobacterium	Full length hit, <7e-19, Genera: Chryseobacterium, Elizabethkingia, Empedobacter Flavobacterium, …	
CJF12_14620_2	Transposase			IS1595 N-term, truncated, similar to CJF12_14530
